# Multi-level consistent changes of the ECM pathway identified in a typical keratoconus twin’s family by multi-omics analysis

**DOI:** 10.1186/s13023-020-01512-7

**Published:** 2020-08-31

**Authors:** Xiao-dan Hao, Xiu-nian Chen, Yang-yang Zhang, Peng Chen, Chao Wei, Wei-yun Shi, Hua Gao

**Affiliations:** 1 Institute for Translational Medicine, The Affiliated Hospital of Qingdao University, College of Medicine, Qingdao University, Qingdao, 266021 China; 2 State Key Laboratory Cultivation Base, Shandong Provincial Key Laboratory of Ophthalmology, Shandong Eye Institute, Shandong First Medical University & Shandong Academy of Medical Sciences, Qingdao, 266071 China; 3 Department of Human Anatomy, School of Basic Medicine, Qingdao University, Qingdao, China; 4 Shandong Eye Hospital, Shandong Eye Institute, Shandong First Medical University & Shandong Academy of Medical Sciences, Jinan, 250021 China

**Keywords:** Keratoconus, Multi-Omics analysis, Polygene, ECM

## Abstract

**Background:**

Keratoconus (KC) is a common, degenerative disorder of the cornea, and genetic factors play a key role in its development. However, the genetic etiology of KC is still unclear. This study used the family of twins as material, using, for the first time, multi-omics analysis, to systematically display the changes in KC candidate factors in patients at the DNA, RNA, and protein levels. These can evaluate candidate pathogenic factors in depth and lock onto pathogenic targets.

**Results:**

The twins in this study presented classic phenotypes, clear diagnoses, complete case data, and clinical samples, which are excellent materials for genetically studying KC. Whole-exome sequencing was conducted on both the twins and their parents. Transcriptome sequencing was conducted on proband’s and health individual’s primary human corneal fibroblast cells. Quantitative Real-time PCR and western blot were used to validate the differential gene expressions between the proband and controls. By integrating genomics, transcriptome, and protein level data, multiple consecutive events of KC were systematically analyzed to help better understand the molecular mechanism and genetic basis of KC. The results showed that the accumulation of rare, micro-effect risk variants was the pathogenic factor in this Chinese KC family. Consistent changes in extracellular matrices (ECMs) at the DNA and RNA levels suggested that ECM related changes play a key role in KC pathogenesis. The major gene variants (*WNT16*, *CD248*, *COL6A2*, *COL4A3* and *ADAMTS3*) may affect the expression of related collagens or ECM proteins, thus reducing the amount of ECM in corneas and resulting in KC.

**Conclusions:**

This study, the first to explore the genetic etiology of KC via multi-omics analysis under the polygenetic model, has provided new insights into the genetic mechanisms underlying KC and an effective strategy for studying KC pathogenesis in the future.

## Background

Keratoconus (KC) is a complex, genetically heterogeneous, multifactor degenerative disorder of the cornea with a worldwide prevalence of approximately 1:2000 [1–4]. It is characterized by corneal ectasia, thinning, and cone-shaped protrusion, resulting in reduced vision, irregular astigmatism, and corneal scarring [1–4]. Because of the unclear pathogenesis and limited availability of medical treatments, KC becomes a significant clinical problem worldwide and a leading indication for corneal transplantation [5]. Previous studies have shown that genetic factors are involved in the KC development [2, 6, 7], and, thus, KC has a clear genetic tendency. About 6–10% of KC patients have a family history of KC, and its incidence in patients’ immediate relatives is 60 times higher than the incidence in people who are not immediate relatives of KC patients [8]. The genetic mode for KC development is autosomal dominant inheritance with incomplete exodominant or recessive inheritance [7, 8]. KC studies among twins show that monozygotic twins display higher genetic consistency than heterozygotic twins, which proves that genetic factors have an important contribution towards the pathogenesis of KC [8, 9].

Therefore, scholars have conducted much research on the genetic causes of KC. So far, more than 30 candidate genes and regions have been screened and identified by genome-wide linkage analysis, whole-exome sequencing (WES), or candidate gene sequencing [7, 8, 10–13]. However, very few of these genes have been assessed for rare variation in keratoconus more broadly [14–18]. The mutation rate of these genes in the KC population were very low, and even cannot be detected in many populations [14–21]. The existing candidate genes can only explain a small proportion of the incidence of KC. Thus, the cause of KC in a large number of patients is still unknown [16, 21, 22].

The screening of susceptible loci is another important method for exploring the genetic causes of complex diseases. Although some KC-related loci have been found, many of them cannot be validated in other populations or in wider ranges of disease populations [17, 23, 24]. Most of the common single-nucleotide polymorphisms (SNPs) that have been located are marker loci rather than pathogenic mutations, which can only explain a small part of the heritability of KC. Therefore, these associated SNPs have a limited effect on the prediction of disease risk but have not achieved ideal results. Due to the genetic heterogeneity and population differences among KC patients, the genetic cause of most cases has not been effectively solved, and the pathogenesis underlying the genetic mutation is still unclear. This has become the bottleneck of KC genetic etiology research, so it is very important to find a breakthrough point to explore the key pathogenic genes, and the common pathogenesis, of KC.

Twins are excellent materials for researching human traits and disease genetics. This study chose a family with KC monozygotic twins. The identical clinical symptom of the twins indicated the existence of genetic factors in the family. This provided precious samples for the study of the genetic mechanism of KC. Multi-omics analysis is an effective strategy for studying pathogenesis in complex diseases [25–27], but it has not been used to explore the KC pathogenesis. To further explore the genetic etiology of KC and reveal its pathogenesis, this study used a family with twins as material – for the first time through the joint analysis of multi-omics – to explore the candidate pathogenic genes of KC, as well as the pathogenic mechanisms underlying the mutations.

## Results

### Clinical features

**Fig. 1:**
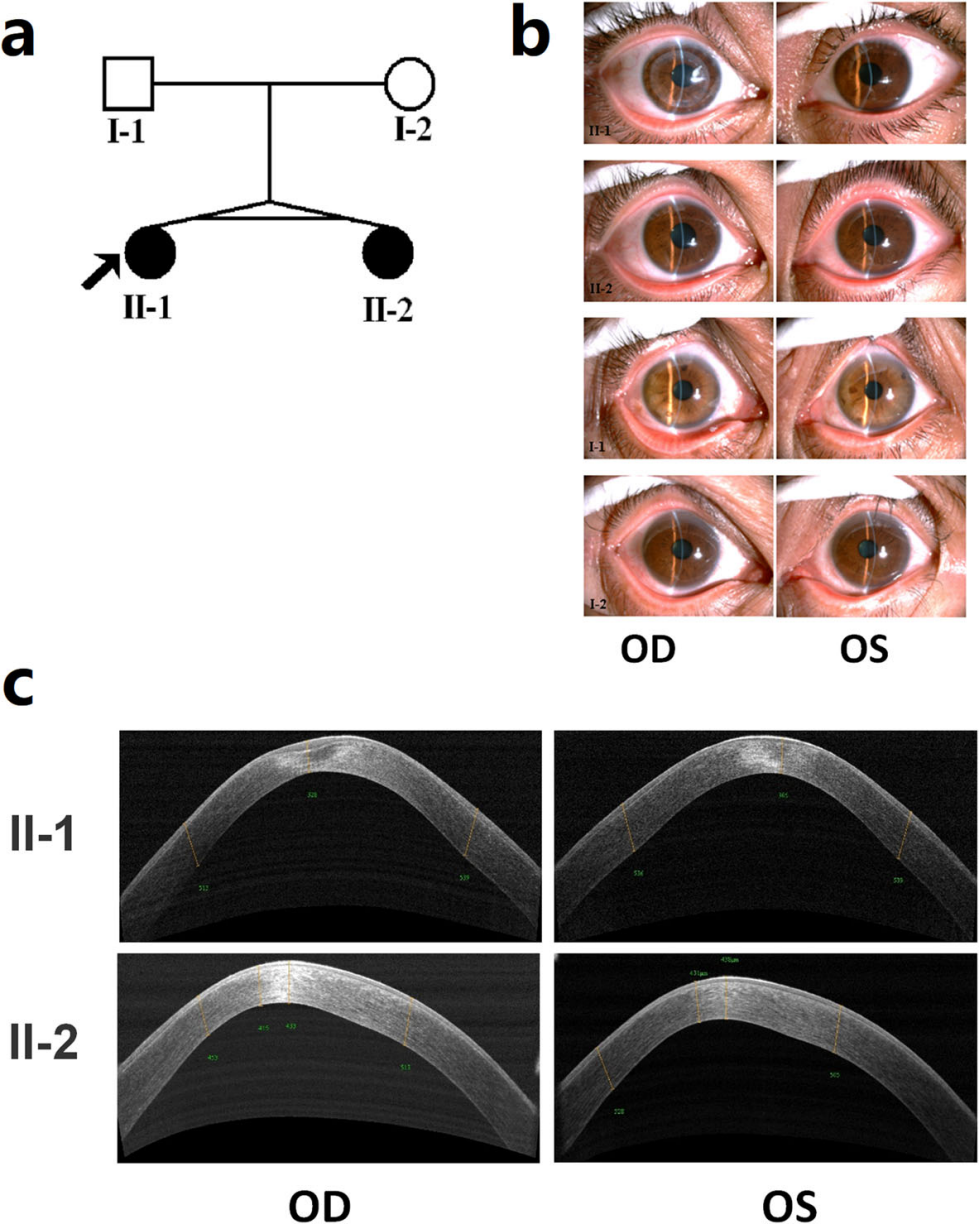
Pedigree and patients’ cornea OCT of the family of twins with KC. **a** Pedigree of the twins’ family. The proband is indicated by an arrow. **b** Slit lamp photographs of the twins’ family. The OD of II-1 was the post-operative photograph, and the rest were the pre-operative photographs. **c** OCT of patients’ corneas. The results showed high central corneal curvature and thinning of the corneal thickness in both eyes. OD, right eye. OS, left eye

**Fig. 2:**
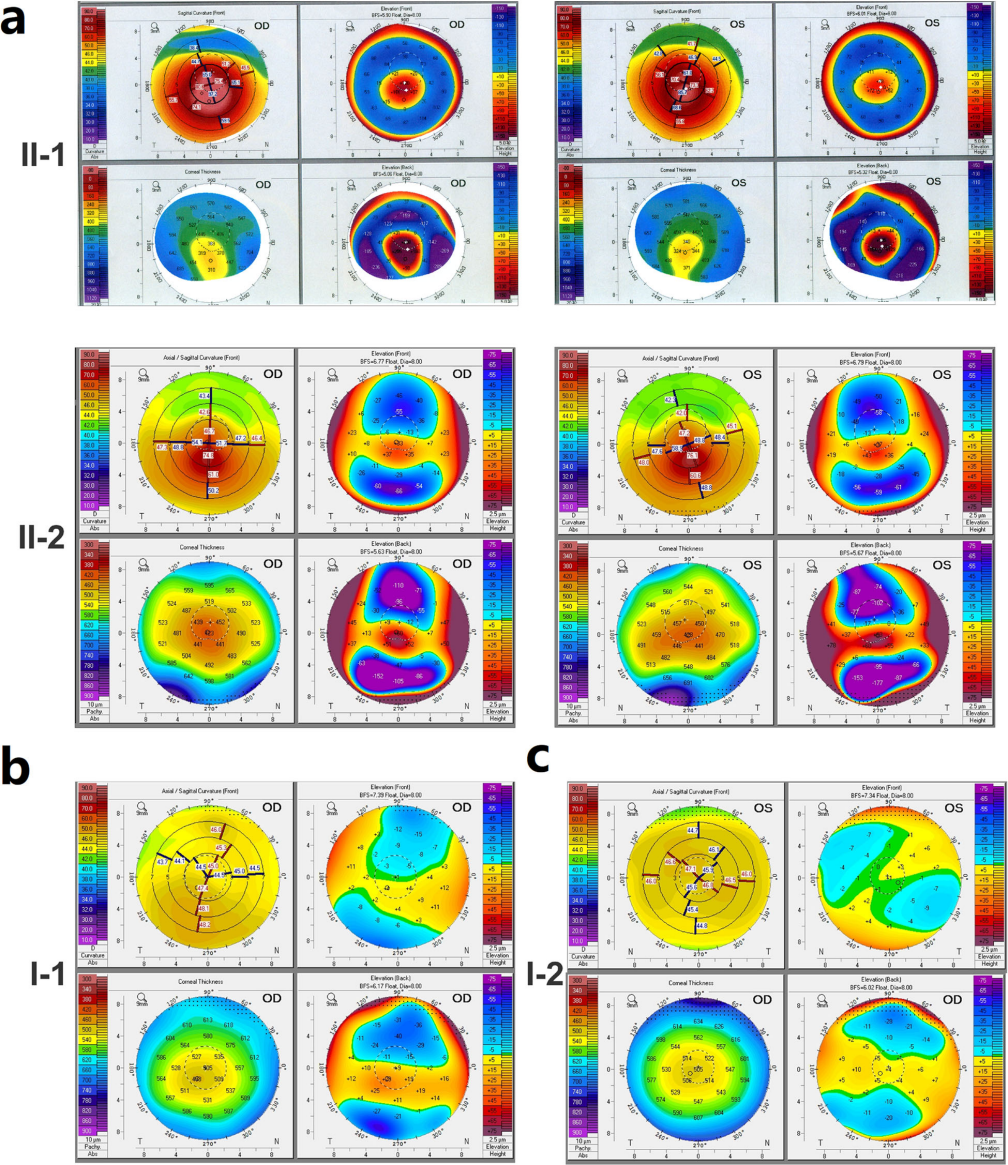
Corneal topographies of the twins’ family. **a** Corneal topographies (including curvature, corneal thickness, front elevation, and back elevation) of the twins (KC patients). **b** Risk manifestations of the twins’ father. **c** Risk manifestations of the twins’ mother. OD, right eye. OS, left eye

The pedigree of this family is shown in Fig. 1a. The proband individual (II-1) and her twin sister (II-2), aged 28, were born from an uneventful pregnancy. The proband patient’s eyes had been ametropic since childhood, and her vision could not be corrected before half a year of hospitalization. She presented to the hospital for medical treatment and was diagnosed with bilateral KC. The vision of the proband right eye (OD) is 0.02 (0.3 × − 8.50DS), and the vision of the left eye (OS) is 0.02 (0.4 × − 8.00DS). Both her eyes have corneal ectasia, thinning, superficial scarring, and a cone-shaped protrusion with Vogt’s striae, Fleischer’s ring, and Munson’s sign (Fig. 1b-c). The results of anterior segment optical coherence tomography (OCT) showed that the thinnest corneal thickness of the left eye is 328 μm, and that of the right eye is 365 μm (Fig. 1c). The proband patient’s signs of videokeratography showed typical KC characteristics, including max corneal curvature >90D, thinnest corneal thickness < 300 μm, max front elevation > 120 and max back elevation > 200 (Fig. 2a).

Clinical examination results showed that the proband individual’s twin sister was a KC patient too. Her slit lamp photographs (Fig. 1b), OCT (Fig. 1c) and videokeratography (Fig. 2a) results showed her to have similar symptoms to her proband twin, but the development process was lighter and slower. Their parents’ symptoms were not great enough to diagnose KC, but they did present some subclinical manifestations. For example, the father’s (I-1) signs of videokeratography showed that his max corneal curvature was 48D; his max back elevation was 29; and his thinnest corneal thickness was 481 μm (Fig. 2b, Additional file 1: Table S1). The mother’s (I-2) signs of videokeratography showed that her max corneal curvature was 47D, and her thinnest corneal thickness was 502 μm (Fig. 2c, Additional file 1: Table S1). The remaining detailed clinical informations for the family are shown in Additional file 1 (Table S1). The clinical manifestations in this family were complex and incomplete consistent with the characteristics of Mendelian disease, which suggested that the inheritance pattern in the family might be polygenetic.

### Exome sequencing detects candidate gene variants under different genetic models

**Fig. 3:**
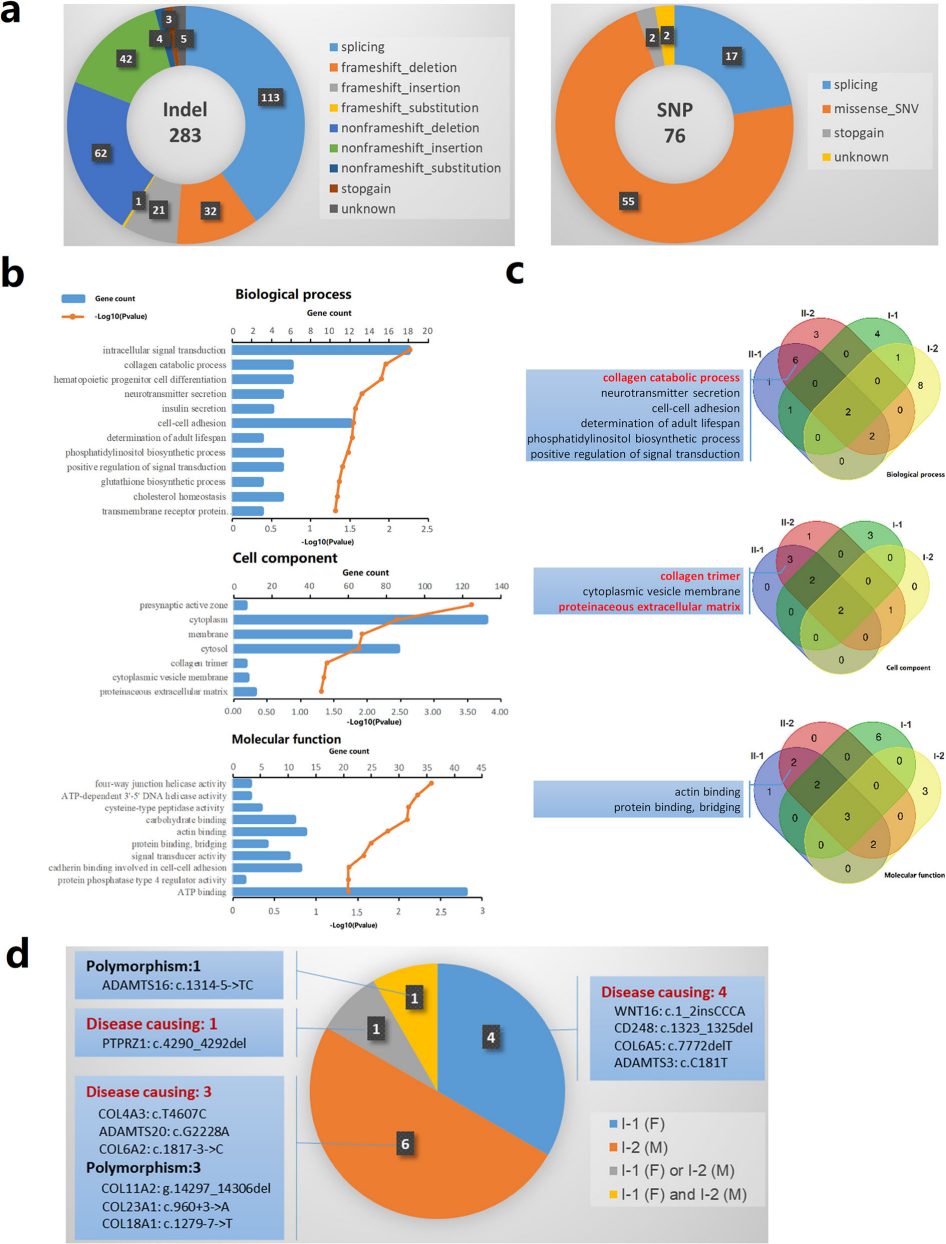
Mutations detected by whole exome sequencing. **a** The detailed mutation types and distribution of the mutations in the twin patients retained after filtering. **b** GO analysis results of all mutations retained. **c** The specific GOs of the twin patients compared to their parents. The GOs having similar functions with the reported pathogenesis of KC are indicated by red color. **d** Function prediction and sources of 12 candidate gene mutations under the polygenetic model

To reveal the underlying genetic defect in this family, the researchers conducted WES on four core members: the twins and their parents. The average sequencing depth and coverage of this analysis were 61.1× and 99.75%, respectively. With the data analysis and variants, as well as the filtering strategy described in the methods section, 283 indels and 76 SNPs of the twin patients were retained. Details of the variation types and distributions are shown in Fig. 3a. The gene ontology (GO) analysis results of all variants were shown in Fig. 3b. The researchers filtered the candidate gene variants under different genetic models, as described in the methods section, and the results were shown in Table 1. All the candidate gene variants obtained by WES were confirmed by Sanger sequencing (Additional file 1: Figure S1). Given the characteristics of the family’s pedigree (Fig. 1a), homozygous, compound heterozygous, or de novo variations were considered the first to be considered as candidate causal variations.

**Table 1:** The candidate gene variants under different genetic models

Gene	Mutation types	Nucleotide change	Amino acid change	Mutation Taster	Source	Genetic models
PKDREJ	missense_SNV	NM_006071.1:c.2708 T > C	NP_006062.1:p.(Ile903Thr)	polymorphism	I-1(F)	autosomal recessive
PKDREJ	missense_SNV	NM_006071.1:c.1780C > G	NP_006062.1:p.(Leu594Val)	disease causing	I-2(M)	
FGA	missense_SNV	NM_000508.4:c.709 T > C	NP_000499.1(LRG_557p1):p.(Phe237Leu)	polymorphism	de novo	de novo
WNT16	frameshift_insertion	NM_016087.2:c.1_2insCCCA	NP_057171.2:p.(Met1?)	disease causing	I-1(F)	polygenic
CD248	nonframeshift_deletion	NM_020404.2:c.1326_1328del	NP_065137.1:p.(Ser443del)	disease causing	I-1(F)	
COL6A5	stopgain	NM_001278298.1:c.7772del	NP_001265227.1:p.(Leu2591Ter)	disease causing	I-1(F)	
ADAMTS3	missense_SNV	NM_014243.2:c.181C > T	NP_055058.2:p.(Leu61Phe)	disease causing	I-1(F)	
COL4A3^a^	missense_SNV	NM_000091.4:c.4607 T > C	NP_000082.2(LRG_230p1):p.(Ile1536Thr)	disease causing	I-2(M)	
ADAMTS20	missense_SNV	NM_025003.4:c.2228G > A	NP_079279.3:p.(Gly743Glu)	disease causing	I-2(M)	
COL6A2	splicing	NM_001849.3:c.1817-3dup	NP_001840.3(LRG_476p1):p.?	disease causing	I-2(M)	
COL11A2	splicing	NM_080680.2:c.1819-18_1819-9del	NP_542411.2:p.?	polymorphism	I-2(M)	
COL23A1	splicing	NM_173465.3:c.960 + 3dup	NP_775736.2:p.?	polymorphism	I-2(M)	
COL18A1	splicing	NM_030582.3:c.1279-7dup	NP_085059.2:p.?	polymorphism	I-2(M)	
PTPRZ1	nonframeshift_deletion	NM_002851.2:c.4290_4292del	NP_002842.2(LRG_1387p1):p.(Asp1431del)	disease causing	I-1(F) or I-2(M)	
ADAMTS16	splicing	NM_139056.3:c.1314-6_1314-5dup	NP_620687.2:p.?	polymorphism	I-1(F) and I-2(M)	

**Notes:** *F* proband’s father, *M* proband’s mother, *FPKM* Fragments per kilobase of exon model per million mapped fragments, *N-HCF* normal HCF cells

^a^reported candidate gene

First, the researchers filtered the remaining variations with the reported candidate genes and found a missense variant in *COL4A3* (NM_000091.4:c.4607 T > C, p. I1536T). The variant c.4607 T > C (p. I1536T) was absent from 1000 Genomes (1000G) but was recorded in the ExAC database (1 heterozygous). Functional prediction under different tools all showed that this variant was “damaging” (SIFT, MutationTaster) or possibly damaging (PolyPhen2). However, this variant was not cosegregation with the phenotype of the family (Fig. 1a, Table 1). According to the ACMG (American College of Medical Genetics and Genomics) standards and guidelines [28], c.4607 T > C in *COL4A3* (PM2, PP2, PP3 and BS4) was uncertain significance, indicating that it was not the cause or not the only cause of the disease.

Of all retained variants, only the *PKDREJ* gene with compound heterozygous variants (NM_006071.1:c.2708 T > C, p.I903T; c.1780C > G, p.L594V) was identified under the autosomal recessive model (Table 1). c.2708 T > C (p.I903T) was absent from 1000 Genomes (1000G) but was recorded in the ExAC database. It was considered a “polymorphism” as predicted by MutationTaster and PolyPhen2. c.1780C > G (p.L594V) was recorded in the 1000G (rs533899886) but had a very low allele frequency (< 0.0002). Functional prediction under different tools all showed that this variant was “damaging” (SIFT, MutationTaster) or possibly damaging (PolyPhen2). According to the ACMG standards and guidelines, variants in *PKDREJ* (PM2, PP1) were uncertain significance.

To detect the de novo variations, the researchers compared the sequencing results from the twins to those of their parents. Three genes with de novo variants were identified, but only one variation in the *FGA* gene (NM_000508.4:c.709 T > C, p.F237L) was found in the functional region and caused amino acid changed (Table 1). This variant was found neither in the ExAC nor the 1000G databases. However, it was considered a “polymorphism,” as predicted by MutationTaster and PolyPhen2, indicating that it might be a benign variation. According to the ACMG standards and guidelines, variant in *FGA* (PS2, PM2, PP1) was likely pathogenic with a contradiction (BP4).

The clinical manifestations of this KC family were incomplete consistent with the characteristics of Mendelian disease. The reported candidate gene variation (*COL4A3*: c.4607 T > C) cannot fully explain the cause of the disease. All of these suggested that the twins’ pathogenesis was probably due to multiple micro-effect variants provided by their parents. Therefore, the researchers also analyzed the variations under the polygenic genetic model. Because of the combination of parental risk variations, these twin patients were likely to have some significant different GO enrichments compared to their parents, which suggested the possible causes of the disease in this family. The specific GOs of the twin patients are shown in Fig. 3c, including six biological processes, three cell components, and two molecular functions. In these specific GOs, one biological process (the collagen catabolic process) and two cell components (collagen trimer and the proteinaceous extracellular matrix (ECM)) function similarly to the reported KC pathogenesis. Finally, 12 gene variants belonging to specific GOs with similar functions were selected as the candidate genes under the polygenetic model (Fig. 3d). Six of these came from the mother (including the c.4607 T > C in reported gene *COL4A3*), four from the father, and one from both, while the origin of the remaining gene was uncertain. Function prediction results from MutationTaster showed that four candidate gene variants were considered “polymorphisms,” and eight were considered “disease causing” (Fig. 3d, Table 1).

### Expression and function analysis further confirmed the pathogenicity of the accumulation of multiple rare, micro-effect risk variants

**Fig. 4:**
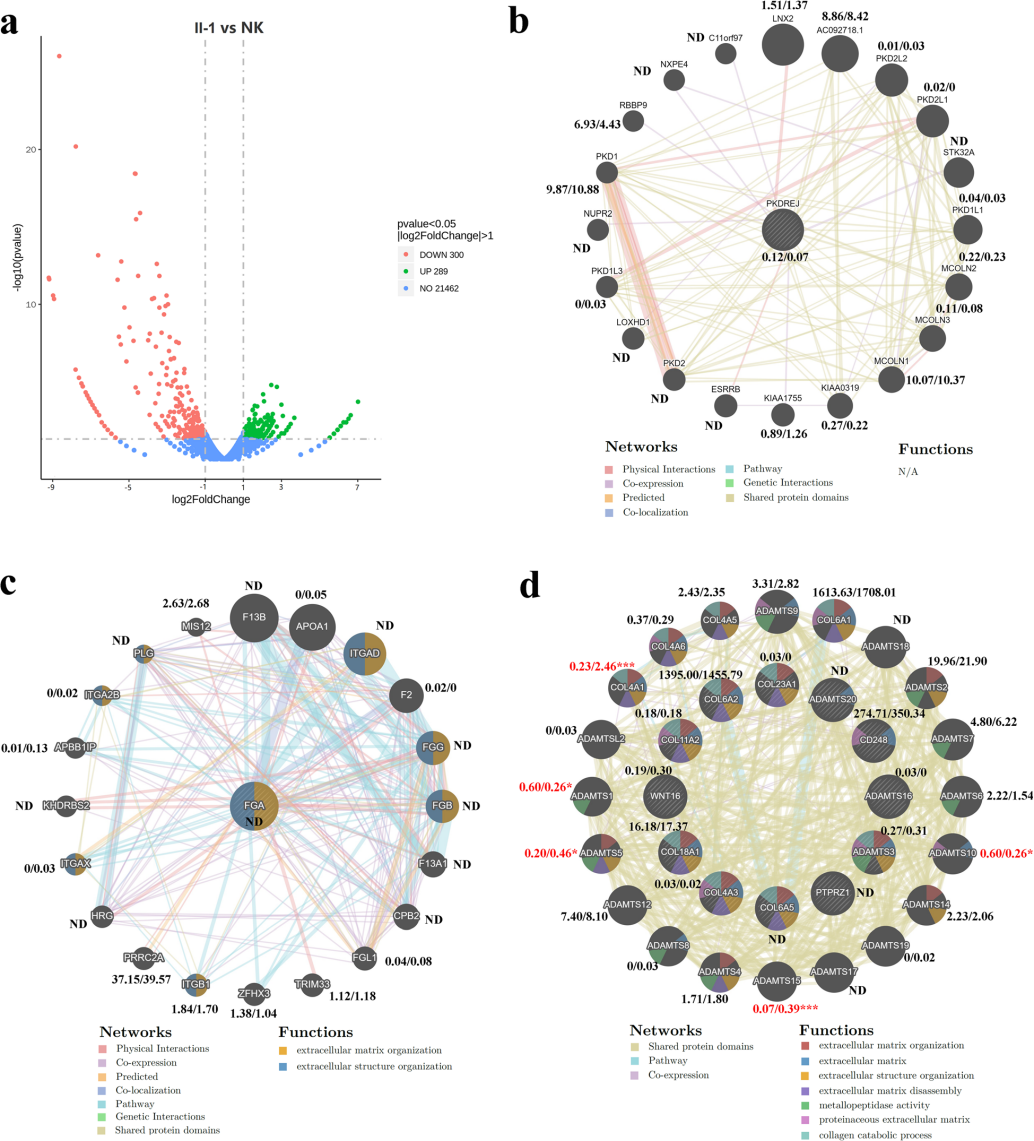
Expression and function analysis of the candidate genes and their top 20 associated genes. **a** 589 differentially expressed genes in KC HCF cells detected by transcriptome sequencing. **b** Expression and function analysis of the *PKDREJ* and its top 20 associated genes. **c** Expression and function analysis of the *FGA* and its top 20 associated genes. **d** Expression and function analysis of the candidate genes (inner ring) under the polygenetic model and their top 20 associated genes (outer ring). FPKM values of each gene in proband (left) and normal (right) HCF cells of were showed. Asterisk represents a significant difference (red color) (*, *p* < 0 .05; **, *p* < 0 .01; ***, *p* < 0 .01). ND, not detected

To further identify the putative pathogenicity of gene variants and detect the KC related gene function and pathway changes, the researchers conducted an expression analysis. Transcriptome sequencing (RNA-seq) on the proband’s primary human corneal fibroblast cells (II-1-HCF) and normal HCF cells were used to assess gene expression changes. All RNA samples were sequenced with 40.2–43.3 million raw reads, and all samples had at least 36 million reads aligned. Sequence reads with multiple alignments were removed during the quality control process. Out of the total 22,051 genes, 10,957 were expressed in the normal HCF cells with FPKM (Fragments Per Kilobase of transcript per Millionfragments mapped) ≥ 1.0, and 11,142 were expressed in patient HCF cells with FPKM ≥1.0. The Pearson correlation coefficient between samples was 0.97. Differential expression analysis identified 589 differentially expressed genes in II-1-HCF cells, with at least a two-fold change and a false discovery rate *p* value ≤0.05 (Fig. 4a, Additional file 2: Table S3).

Then, the expression levels of the candidate genes determined at the DNA level were analyzed. The results indicated whether each candidate gene was expressed in corneal cells, and weather each candidate gene variant affects the expression of itself or expression of its function related genes. *PKDREJ*, detected under the autosomal recessive model with uncertain significance, was essentially not expressed (FPKM = 0.11) in normal HCFs (Fig. 4b). No significant difference was detected in the expression of its top 20 associated genes between the proband and normal HCF cells (Fig. 4b)*.* In addition, *PKDREJ* and its associated genes did not have any same or similar function with the reported pathogenesis of KC. All of these suggest that *PKDREJ* is not the pathogenic gene in the sampled KC family.

Similarly, *FGA*, detected under the de novo mutation model, was not expressed in both the proband and normal HCF cells (Fig. 4c), indicating that it may not be involved in the structure and function of cornea. Although *FGA* and its top 20 associated genes have some similar functions with the reported pathogenesis of KC, many of them was essentially not expressed in HCF cells (Fig. 4c). The expression levels of rest genes also had no significant difference between the proband and normal HCF cells (Fig. 4c). All of these results suggest that *FGA* is not the pathogenic gene in the sampled KC family too.

The FPKM values of the 12 candidate genes detected under the polygenetic model are shown in (Fig. 4d). Among them, 8 candidate genes (*COL23A1*, *CD248*, *ADAMTS16*, *ADAMTS3*, *COL4A3*, *COL18A1*, *WNT16*, and *COL6A2*) were expressed in corneal cells. There was no significant difference in the expression of candidate genes between the proband and normal HCF cells (Fig. 4d). Among their top 20 associated genes, 5 genes (*ADAMTS1*, *ADAMTS5*, *ADAMTS10*, *ADAMTS15*, and *COL4A1*) showed significant differential expression (Fig. 4d). In addition, these candidate genes and their top 20 associated genes were enriched in many similar functions with the reported pathogenesis of KC. These results suggested that these variants did not affect the expression of themselves, but affected the expression of function related genes, then caused the functional changes consistent with the known pathogenesis of KC, which will undoubtedly lead to development of KC. All of these suggested that the accumulation of multiple rare, micro-effect risk variants might be the pathogeny of the sampled KC family.

### Multi-level consistent changes of the ECM pathway identified by multi-omics analysis

**Fig. 5:**
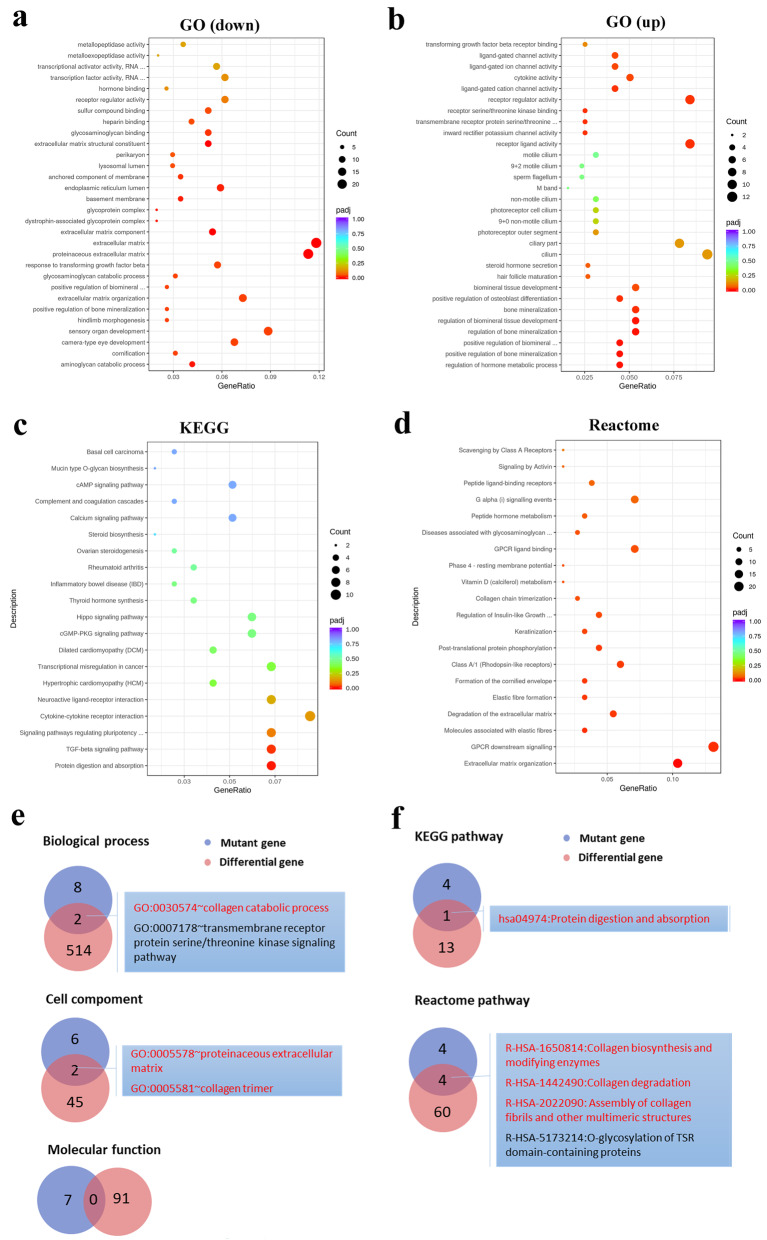
Consistent changes of the ECM pathway identified by multi-omics analysis. **a** Top ten enriched GOs (each category) of 300 downregulated genes. **b** Top ten enriched GOs (each category) of 289 upregulated genes. **c** TOP 20 enriched KEGG pathways of differentially expressed genes. **d** TOP 20 enriched Reactome pathways of differentially expressed genes. **e** The shared GOs of candidate variant genes and differentially expressed genes. The GOs having similar functions with the reported pathogenesis of KC are indicated by red color. **f** The shared KEGG and Reactome pathways of candidate variant genes and differentially expressed genes. The pathways having similar functions with the reported pathogenesis of KC are indicated by red color

In order to further explore the molecular pathogenesis of KC, we performed differential expression analysis and identified 589 differentially expressed genes in II-1-HCF cells, with at least a two-fold change and a false discovery rate *p* value ≤0.05 (Additional file 2: Table S3). There were 300 downregulated and 289 upregulated genes in the proband HCF cells. Gene ontology analysis using the 300 downregulated genes indicated that the top five significant enrichments of the genes coding for proteins were involved in or related to the proteinaceous ECM (adjust *p*-value (padj): 5.56E-09), the ECM (padj: 4.02E-08), the ECM structural constituent (padj: 3.46E-06), the ECM component (padj: 2.49E-05), and the aminoglycan catabolic process (padj: 3.64E-03) (Fig. 5a). Gene ontology analysis using the 289 upregulated genes indicated that the top five significant enrichments of genes coding for proteins were involved in or related to the regulation of the hormone metabolic process (padj: 2.97E-03), the positive regulation of bone mineralization (padj: 3.00E-03), the positive regulation of biomineral tissue development (padj: 3.00E-03), the regulation of bone mineralization (padj: 3.00E-03), and the regulation of biomineral tissue development (padj: 3.00E-03) (Fig. 5b). Kyoto Encyclopedia of Genes and Genomes (KEGG) pathway analysis using all 589 genes identified the enriched pathways, including protein digestion and absorption and the TGF-beta signaling pathway (Fig. 5c). Reactome analysis using all 589 genes indicated the involvement of ECM organization, GPCR downstream signaling, degradation of the ECM, and elastic fiber formation (Fig. 5d).

Combined analysis of exome and transcriptome enrichment revealed their shared GOs and pathways, allowing the researchers to evaluate candidate pathogenic factors in depth and lock onto pathogenic targets. The shared GOs of exome and transcriptome sequencing results are shown in Fig. 5e, including the two biological processes and two cell components. In these shared GOs, 75% (including the collagen catabolic process, collagen trimer, and the proteinaceous ECM) had similar functions with the reported pathogenesis of KC. The combined analysis of the KEGG pathway showed that protein digestion and absorption was significantly enriched both at DNA and RNA levels (Fig. 5f). The combined analysis of the Reactome pathway showed that there were four pathways shared by exome and transcriptome sequencing results, among which 75%, including collagen biosynthesis and modifying enzymes, collagen degradation, and the assembly of collagen fibrils and other multimeric structures, had similar functions with the reported pathogenesis of KC (Fig. 5f). Most GO and pathway enrichments shared at DNA and RNA levels were related to collagen and the ECM, suggesting that these GO and pathway changes might have been etiological—serving as mechanisms of KC in this family.

### Validation of differential gene expressions involved in the ECM pathway

**Fig. 6:**
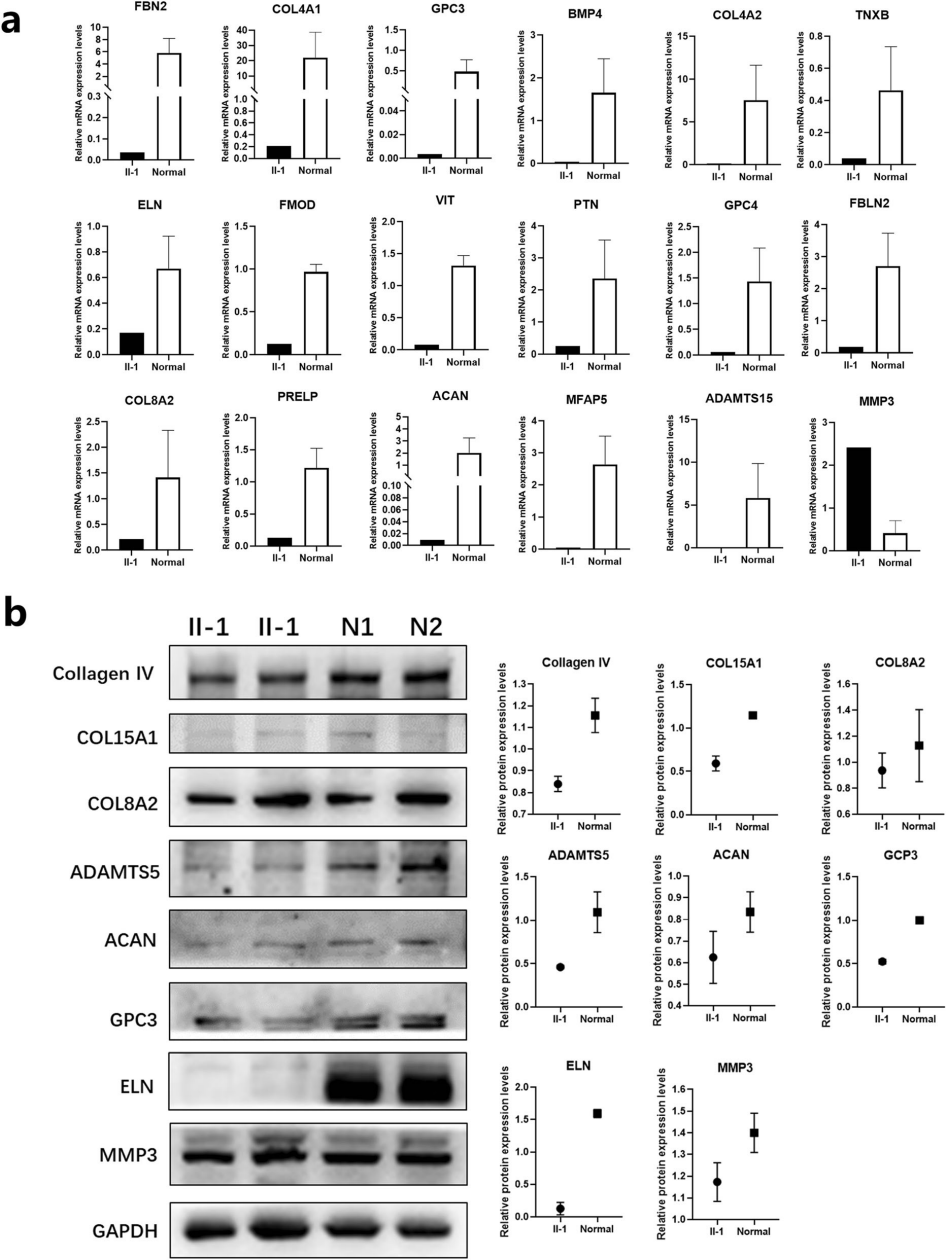
qRT-PCR and western blot validation of differential gene expressions. **a** qRT-PCR results for 18 genes with top *p* values. **b** Western blot results for top eight genes of network nodes

The researchers further validated the differential gene expressions by selecting 18 genes involved in the ECM pathway (*FBN2*, *COL4A1*, *GPC3*, *BMP4*, *COL4A2*, *TNXB*, *ELN*, *FMOD*, *VIT*, *PTN*, *GPC4*, *FBLN2*, *COL8A2*, *PRELP*, *ACAN*, *MFAP5*, *ADAMTS15*, and *MMP3*) with top *p* values. These were compared to the RNA-Seq results with RNA expression in the proband and in three normal HCF cells using qRT-PCR. Among the genes tested, 16 genes’ expression levels (*FBN2*, *COL4A1*, *BMP4*, *COL4A2*, *TNXB*, *ELN*, *FMOD*, *VIT*, *PTN*, *GPC4*, *FBLN2*, *PRELP*, *ACAN*, *MFAP5*, *ADAMTS15*, and *MMP3*) in the proband cells were consistently lower or higher than in all controls (Fig. 6a), which were consistent with this study’s RNA-Seq findings.

To further mine for key pathogenic factors, the authors analyzed the intermediary network of all related genes, including 12 candidate variant genes and 32 differentially expressed genes in shared enrichments. The protein interaction network showed that most of the genes were gathered into two groups (Additional file 3: Figure S2). One group was the collagen family of genes (consisting of six variant genes and five differential genes), and the other was the ADAMTS family of genes (consisting of three variant genes and four differential genes). Genes with multiple network nodes are believed to play an important role in KC pathogenesis. The authors selected the top eight proteins (Collagen IV, COL15A1, COL8A2, ADAMTS5, ACAN, GPC3, ELN and MMP3) of the network nodes for western blot validation. The results showed that five proteins’ levels (Collagen IV, COL15A1, ADAMTS5, GPC3 and ELN) in the proband cells were consistently lower than in all controls (Fig. 6b), which were consistent with this study’s RNA-Seq findings.

## Discussion

KC is a complex, genetically heterogeneous, multifactorial, degenerative disorder of the cornea [1–3]. Previous reports have detected more than 30 candidate genes and regions associated with KC [7, 8]. However, because of the disease’s genetic heterogeneity and specificity, its genetic causes in most patients have not been effectively solved. In addition, the pathogenesis, under the coverage of genetic mutations, remains unclear. This has become the bottleneck of KC genetic etiology research, so it is very important to find a breakthrough point to explore the key pathogenic genes of KC and common pathogenesis. Therefore, this study used the family of twins as material, using, for the first time, multi-omics analysis, to systematically display the changes in KC candidate factors in patients at the DNA, RNA, and protein levels. These can evaluate candidate pathogenic factors in depth and lock onto pathogenic targets. By integrating genomics, transcriptome, and protein level data, the authors systematically analyzed multiple consecutive events in KC, which would provide a better understanding of the molecular mechanisms and genetic bases of complex characters in KC.

The twins in this study presented classic phenotypes, clear diagnoses, complete case data, and clinical samples, which are excellent materials for genetically studying KC. The clinical symptoms in these twins were very typical and consistent, including corneal ectasia, thinning, superficial scarring, and a cone-shaped protrusion with Vogt’s striae, Fleischer’s ring, and Munson’s sign. Their parents’ symptoms were not pronounced enough to diagnose KC, but they did present some subclinical manifestations, including larger corneal curvatures, back elevation, and thinner corneal thickness compared to normal, unrelated individuals [29–31]. These complex clinical manifestations in this family supported the hypothesis of a polygenic inheritance pattern.

WES was conducted on both the twins and their parents. In addition to the traditional Mendelian genetic model, the polygenetic model and the de novo mutation model were also selected to screen for pathogenic genes at the DNA level, which greatly improved the possibility of detecting candidate gene mutations. Under the autosomal recessive model, the researchers identified the *PKDREJ* gene with rare compound heterozygous variants. However, one of the variants was considered a “polymorphism,” as predicted by different tools. *PKDREJ* (full name: polycystin family receptor for egg jelly) is located on chromosome 22q13.31 and encodes a member of the polycystin protein family, which may have a central role in human fertilization [32]. A previous study has reported that *PKDREJ* has a very restricted expression pattern, and its transcript was found exclusively in testes [32]. The present transcriptome sequencing results also showed that this gene is essentially not expressed in the normal human cornea. All of this suggests that *PKDREJ* is not the pathogenic gene in the sampled KC family. The authors also identified the *FGA* gene with a de novo variant in the functional region. Considering the benign functional prediction results and the absence of expression in HCF cells, the FGA gene was also eliminated as a candidate pathogenic gene in the sampled family.

Due to the combination of parental variants, the sampled twin patients received three specific collagen or ECM related GO enrichments compared to their parents. Changes in collagen and the ECM have been reported as being involved in KC [3, 33–36]. The accumulated variants of these three GOs may affect the normal function of collagens or ECMs, resulting in KC development. After screening specific and reported pathogeneses, which were functionally similar to the GOs in the sampled patients, the researchers detected 12 candidate gene variants under the polygenetic model. As predicted, some of these variants came from the twins’ mother and some from their father. Among them, just five genes (*WNT16*, *CD248*, *COL6A2*, *COL4A3*, and *ADAMTS3*) were predicted as “disease causing” and expressed in normal HCF cells. Among them, *COL4A3* is a reported candidate genes of KC [37, 38]. However, its variant was not cosegregation with the phenotype of the family, indicating that this variant cannot fully explain the cause of the disease. All of these suggested that the accumulation of these five gene variants were the key pathogenic factors at the DNA level.

The transcriptome sequencing results also showed that there were 589 differentially expressed genes in II-1-HCF cells. For all the differentially expressed genes, in the top five enriched GOs, four were involved in or related with the ECM, and, in the top 5 enriched pathways, four were involved in or related with the ECM or elastic fibers. These results were consistent with previous studies, indicating that the expression changes of the genes related to the ECM play an important role in the KC pathogenesis and development [34, 35]. In addition, the GPCR downstream signaling pathway and the regulation of the hormone metabolic process were the next most significantly enriched pathways and GOs. This suggested that they may also be involved in KC development. Hormone changes have been reported as affecting corneal structure and KC [3]. However, no other literature or evidence supports the relationship between the GPCR downstream signaling pathway and KC.

Multi-omics analysis is an effective strategy for studying the pathogenesis of complex diseases [25–27]. To further explore the genetic etiology of KC and reveal its pathogenesis, the researchers conducted a combined analysis of exome and transcriptome sequencing results. The shared GOs and pathways of exome and transcriptome changes were the possible causes of KC pathogenesis. Combined analysis of exome and transcriptome enrichment found four shared GOs and four Reactome pathways. 75% of these GOs, including the collagen catabolic process, collagen trimer, and the proteinaceous ECM, were involved in or related to collagen or the ECM. 75% of these Reactome pathways, including collagen biosynthesis and modifying enzymes, collagen degradation, and the assembly of collagen fibrils and other multimeric structures, were also involved in or related to collagen. These suggested that these GO and pathway changes might be etiological—serving as KC mechanisms in the sampled family.

The protein interaction network of genes in shared enrichments showed that most of the genes were gathered into two groups. One group was the collagen family of genes, and the other was the ADAMTS family of genes. In the collagen family genes, *COL6A2* and *COL4A3* were expressed in normal HCF cells and also had “disease causing” variants in patients. The changed COL6A2 and COL4A3 protein may also have affected the expression of other related collagen proteins, such as COL4A1, COL4A2, COL15A1 etc. (verified both by qRT-PCR and western blot), thus reducing the number of ECMs within the patients’ corneas. In the ADAMTS family of genes, only *ADAMTS3* was expressed in normal HCF cells (FPKM values> 0.3) and also had “disease causing” variant in the sampled patients. The mutated ADAMTS3 protein may also have affected the expression of other ADAMTS members (such as *ADAMTS10*) and, thus, reduced their substrates or the expressions of other related proteins (also validated by qRT-PCR), including aggrecan (ACAN: ECM components which could protect collagen fibrils from degradation by collagenases) [39], TNXB (ECM glycoproteins) [40], FMOD (interaction proteoglycans for ECM assembly), and PRELP (binding protein in ECM) (https://www.uniprot.org/). In addition, there was a group of genes interacting with both the collagen family and the ADAMTS family in the protein interaction network. All of the downregulated expression genes (validated by qRT-PCR or western blot) in this group were also involved or related with ECM, such as ELN (elastic fibers: part of the ECM) [41], FBLN2 (an ECM protein), MFAP5 (a component of microfibrils of the ECM), and FBN2 (a component of microfibrils for elastic fiber assembly) (https://www.uniprot.org/). Collagen is the main component of cornea stroma, and ECM changes are an important pathogenesis factor for KC [33, 34]. These changes undoubtedly play a major role in stromal thinning and Bowman’s layer/basement membrane breaks, which are characteristic of KC corneas.

## Conclusions

To the best of the authors’ knowledge, this study is the first to explore the candidate pathogenic genes and pathogenic mechanisms of KC by multi-omics analysis. Multi-omics analysis can realize and display the expression levels of candidate genes determined at the DNA levels, analyze the different expressions of mutated genes at the RNA level, and facilitate the enrichment analysis of key genes at the GOs and pathways, etc. Integrating genome, transcriptome, and protein level data allowed the researchers to systematically analyze multiple successive disease events. According to the changes of candidate factors at different levels, candidate pathogenic factors can be mined in depth to lock onto a pathogenic target. Thus, multi-omics analysis is an effective strategy for studying KC pathogenesis. Via multi-omics analysis, the authors identified multiple gene variants (*WNT16*, *CD248*, *COL6A2*, *COL4A3*, and *ADAMTS3*) as candidate genes in the sampled KC family, which suggested that the polygenetic model was the inheritance pattern for this KC and should be considered when detecting pathogenic genes in the future. Multi-level, consistent changes in the ECM pathway in this typical Chinese family with KC twins suggested that these ECM pathway changes play a key role in KC pathogenesis. In addition to collagens, other ECM related changes, such as decreases in aggrecan, glycoproteins, interaction proteoglycans, binding protein, elastic fibers, and microfibrils, also play an important role in KC pathogenesis, which provides new insights into the molecular mechanisms underlying KC and, therefore, must be given more attention. This study extends the existing spectrum of disease-causing genes and further defines the genotype-phenotype correlations. It not just worth for this family, but also provides new insights into the genetic etiology and molecular mechanisms underlying KC, which will provide theoretical basis for the clinical diagnosis, treatment and epidemiological investigation. On the other hand, the new genetic model (the polygenetic model) and research method (multi-omics analysis) also provide an effective strategy for studying KC etiopathogenesis in the future.

## Methods

### Subject recruitment and clinical examination

This study was performed in accordance with the Declaration of Helsinki and approved by the Ethics Committee of Shandong Eye Institute (Qingdao, China). Written informed consent was obtained from all participants (or their guardians). Patients diagnosed with KC were recruited from Qingdao Eye Hospital (Qingdao, China). KC diagnosis was based on clinical examination (corneal stromal thinning, Vogt’s striae, Fleischer’s ring, Munson’s sign, signs of videokeratography, and refractive errors). In total, the family of one pair of monozygotic twins was collected. The pedigree (Fig. 1a) suggested that the inheritance pattern among the twins’ family was autosomal recessive, de novo mutation, or polygene mutations. Peripheral blood samples from all participating individuals were collected in EDTA (Ethylene diamine tet raacetic acid) tubes, and genomic DNA was extracted with a Blood DNA Kit (Tiangen Biotech Co., Beijing, China).

### Whole-exome sequencing (WES) and data analysis

WES was conducted among all members of this family (the twins and their parents) at Novogene (Beijing, China) to identify the KC causal gene. The SureSelect Human All ExonV5 Kit (Agilent Technologies, USA) was used for exome capture, and the IlluminaHiseq 2500 platform (Illumina Inc., San Diego, CA, USA) was employed for the genomic DNA sequencing of the twins and their parents. Then, data analysis was conducted, and filtering was applied, following the filtering strategy described in the previous study [12]. Considering the incidence rate (0.0005) of KC, variants were common (MAF > 1%) in dbSNP, and the 1000 Genomes Project were filtered according to reported guidelines for the interpretation of sequence variants [28, 42].

Given the characteristics of the family’s pedigree, homozygous, compound heterozygous, or de novo variations were considered the first to be considered as candidate causal variations [43]. All candidate variants were submitted to Tolerant (SIFT) [44], PolyPhen-2 [45], and Mutation Taster [46] for functional prediction. At the same time, all other variations among the family members were collected for GO and pathway analysis via the online Database for Annotation, Visualization, and Integrated Discovery (DAVID) software, version 6.8 [47, 48]. By comparing the KC twins with their non-KC parents, the specific GOs and pathways of the patients could be determined. Gene variants belonging to GOs or pathways having similar functions with known KC pathogenesis were selected as the candidate genes for the polygenetic model. Sanger sequencing and genotyping of the candidate genes detected by WES were performed, as described in the previous study [12]. According to these results, the possible genetic model and candidate gene variants in this family were determined at the DNA level.

### Transcriptome sequencing and data analysis

Normal primary human corneal fibroblast (N-HCF) and primary corneal fibroblast cells of II-1 (II-1-HCF) were isolated from normal and proband (II-1) corneas, as described previously [49], and cultured in a DMEM/F12 medium (Corning, USA) containing 10% fetal bovine serum (FBS) (Gibco, USA) at 37 °C with 5% CO_2_. For the isolation of normal human corneal fibroblast cells, corneal tissues were obtained from the Eye Bank at Shandong Eye Institute. Corneal epithelium and endothelium were removed by digestion with 50 mg/ml dispase II (Roche, Switzerland) overnight at 4 °C [49]. The corneal stroma was cut into pieces and incubated 6–8 h at 37 °C in DMEM/F-12 medium containing 1.25 mg/ml collagenase (Invitrogen, USA), until the tissue smeared onto the dish bottom [49]. For the proband, corneal tissues were obtained from Qingdao Eye Hospital, Shandong Eye Institute (Qingdao, China), and the corneal fibroblast cells were isolated as described above immediately after operation. Total RNA was prepared from N-HCF and II-1-HCF samples using the NucleoSpin RNA II System (Macherey-Nagel, Duren, Germany). RNA-seq was also conducted in these two type cells at Novogene (Beijing, China) to identify expression changes in the causal gene. The NEBNext® UltraTM RNA Library Prep Kit (NEB, USA) was used to generate sequencing libraries, and the TruSeq PE Cluster Kit v3-cBot-HS (Illumia) was used to cluster the index-coded samples. Then the library preparations were sequenced on an Illumina Novaseq platform, and 150 bp paired-end reads were generated. FeatureCounts v1.5.0-p3 was used to count the number of reads mapped to each gene. Then FPKMs for each gene were calculated based on the length of the gene and reads count mapped to the gene. The differences in gene expression between proband and normal HCFs was analyzed using the edgeR R package (3.18.1) to determine the specific genes associated with KC, and the biological significance of these specific genes was analyzed by GO and pathway enrichment using the clusterProfiler R package.

### Combined analysis of exome and transcriptome sequencing

The expression levels of the candidate genes, determined at the DNA level, were analyzed and displayed by Excel drawing. These results indicated whether each candidate gene was expressed in corneal cells and whether the expression level of candidate genes in the KC patients’ corneal cells were significantly different compared to normal corneal cells. On the other hand, to determine whether there were corresponding functional changes caused by candidate gene variants in the KC patients, their top 20 associated genes’ expression at the RNA levels were also screened and analyzed. The top associated genes and functions of candidate genes were predicted by GeneMANIA software [50]. Combined analysis of exome and transcriptome sequencing results revealed their shared GOs and pathways, allowing the authors to explore the possible pathogenesis of KC.

### Quantitative real-time PCR

Quantitative Real-time PCR (qRT-PCR) was used to verify the expression differences between related genes. cDNA was synthesized from RNA using a commercial kit (PrimeScript TM RT Reagent Kit (Perfect Real Time); Takara, Dalian, China). Expressions of the KC related genes, determined at the RNA levels, were measured by qRT-PCR and normalized to glyceraldehyde-3-phosphate dehydrogenase (GAPDH). Primer sequences of the genes used for qRT-PCR are shown in Additional file 1: Table S2.

### Western blot

To verify the changes of gene function and pathways in protein levels, western blot analyses were performed, as described previously [12]. First, the researchers collected corneal cells from normal individuals and from the family of the KC twins, extracted the total protein using radioimmunoprecipitation assay (RIPA) buffer (Galen, Beijing, China). For each sample, the levels of proteins of interest were normalized to that of GAPDH. Primary antibodies included Collagen IV antibody (ab6586, Abcam), COL15A1 antibody (D162738, Sangon Biotech), COL8A2 antibody (D262652, Sangon Biotech), ACAN antibody (A8536, Abclonal), ADAMTS5 antibody (D260094, Sangon Biotech), ELN antibody (D120588, Sangon Biotech), GPC3 antibody (A1496, Abclonal), MMP3 antibody (ab52915, Abcam), and anti-GAPDH antibody (KC-5G5, Kangchen, Shanghai, China).

## Supplementary information

**Additional file 1: Table S1.** Clinical characteristics of members of the KC twins’ family. **Table S2.** The primers for Quantitative Real-time PCR (qRT-PCR). **Figure S1.** Sanger sequencing results of candidate gene variants.

**Additional file 2: Table S3.** Five hundred eighty-nine differentially expressed genes in II-1-HCF cells with at least 2-fold change and false discovery rate *p* value ≤0.05.

**Additional file 3: Figure S2.** Protein interaction network between candidate variant genes (inner ring) and differential expression genes (outer ring) in shared enrichments.

## Data Availability

The datasets used and/or analysed during the current study are available from the corresponding author on reasonable request.

## Abbreviations

KC: Keratoconus
ECMs: Extracellular matrices
WES: Whole-exome sequencing
SNPs: Single-nucleotide polymorphisms
OD: Right eye
OS: Left eye
OCT: Optical coherence tomography
GO: Gene ontology
1000G: 1000 Genomes
HCF: Primary human corneal fibroblast cells
FPKM: Fragments Per Kilobase of transcript per Millionfragments mapped
padj: adjust *p*-value
KEGG: Kyoto Encyclopedia of Genes and Genomes
qRT-PCR: Quantitative Real-time PCR
RNA-Seq: Transcriptome sequencing
EDTA: Ethylene diamine tet raacetic acid
DAVID: Database for Annotation, Visualization, and Integrated Discovery
FBS: Fetal bovine serum
GAPDH: Glyceraldehyde-3-phosphate dehydrogenase
RIPA: Radioimmunoprecipitation assay
