# Supplementary material for: Multi-level consistent changes of the ECM pathway identified in a typical keratoconus twin’s family by multi-omics analysis
Source: Orphanet J Rare Dis. 2020 Aug 31;15:227. doi: 10.1186/s13023-020-01512-7 (PMC7457807; doi:10.1186/s13023-020-01512-7)
Supplement: Supplementary file 1 — Additional file 1: Table S1. Clinical characteristics of members of the KC twins’ family. Table S2. The primers for Quantitative Real-time PCR (qRT-PCR). Figure S1. Sanger sequencing results of candidate gene variants. [file 13023_2020_1512_MOESM1_ESM.pdf]

Table S1. Clinical characteristics of members of the KC twins' family.

Sample name	Age	Phenotype	OD (Preoperative)					OS (Preoperative)					Other clinical symptoms
			CVA	Max front elevation	Max back elevation	Max curvature	CCT (μm)	CVA	Max front elevation	Max back elevation	Max curvature	CCT (μm)	
II-1	28	KC	0.3	120	215	97.2	289	0.4	78	195	85.7	291	Vogt's striae (+), Fleischer ring (+), Monson's sigh (+)
II-2	28	KC	0.3	37	60	74.8	422	0.4	43	63	76.1	427	Vogt's striae (+), Fleischer ring (+)
I-1	56	Normal	1.0	4	29	48.2	493	1.0	5	25	47.1	481	none
I-2	55	Normal	1.0	1	5	46.9	502	1.0	1	3	47.1	506	none

Notes: KC, keratoconus; CVA, corrected visual acuity; OD, right eye; OS, left eye; CCT, Thinest central corneal thichness.

Table S2. The primers for Quantitative Real-time PCR (qRT-PCR)

Gene name	Forwad 5'-3'	Reverse 5'-3'
FBN2	GTTTTCTGCCAGTCATCCAGC	AGCTGCTTTGGCTTCGATCT
COL4A1	ACCAAAAGGTGACAAGGGTG	CTGGTTTTCCGGGTTCACCT
GPC3	GCTGACCACCACTATTGGCA	GTTCCCTTCTTCGGCTGGAT
BMP4	GGAGCTTCCACCACGAAGAA	GGAAGCCCCTTTCCCAATCA
COL4A2	CTGCCACTACTACGCCAACA	CCGGCTCACAGGTTCTTCAT
TNXB	GACCAGAGCAAGATCCTCATCTCA	AACTGGTGGTCACGTCAGTCA
ELN	TTTATCCAGGGGCTGGTCTC	AAAGGTAACTGCGGGGAAGG
FMOD	GGGCATACAACCCTCTGCTT	GTGCTCCCCAGCCAAACTAT
VIT	GATGCAGGAAAGCCTAAGGGA	CGGCTTTGACATCGCAGTTG
PTN	GAGCTGAGTGCAAGCAAACC	GCTCGCTTCAGACTTCCAGT
GPC4	TCGCTTCAGACCACATCACC	TCGGAAGGGAGGACCAGAAT
FBLN2	GATGGCGTGTCCTGTGAAGA	TGAGTGCCTTGTAGCAGTGG
COL8A2	TCAAGCGGAGTGGATAAGGC	ACCGAATGACCCCAGTTCAC
PRELP	AGGTGCATCACCTGGATCAT	CGGGTCTTGGTCGTCTTGTT
ACAN	GTGGAGGAGTGGATCGTGAC	TCTGGTTTTCTGGCTCGGTG
MFAP5	TATTTTGCTGGCTTGCCCGA	TCCTCATGCTTTAGCCCCAG
ADAMTS15	CTCCGTGGGGAAGATGACAC	TTGTGCAAGACAGAGGGTCC
MMP3	AGGCAAGACAGCAAGGCATA	AGGTTCATGCTGGTGTCCTC
GAPDH	ATGCTGGCGCTGAGTACGT	AGCCCCAGCCTTCTCCAT

Figure S1. Sanger sequencing results of candidate gene varints

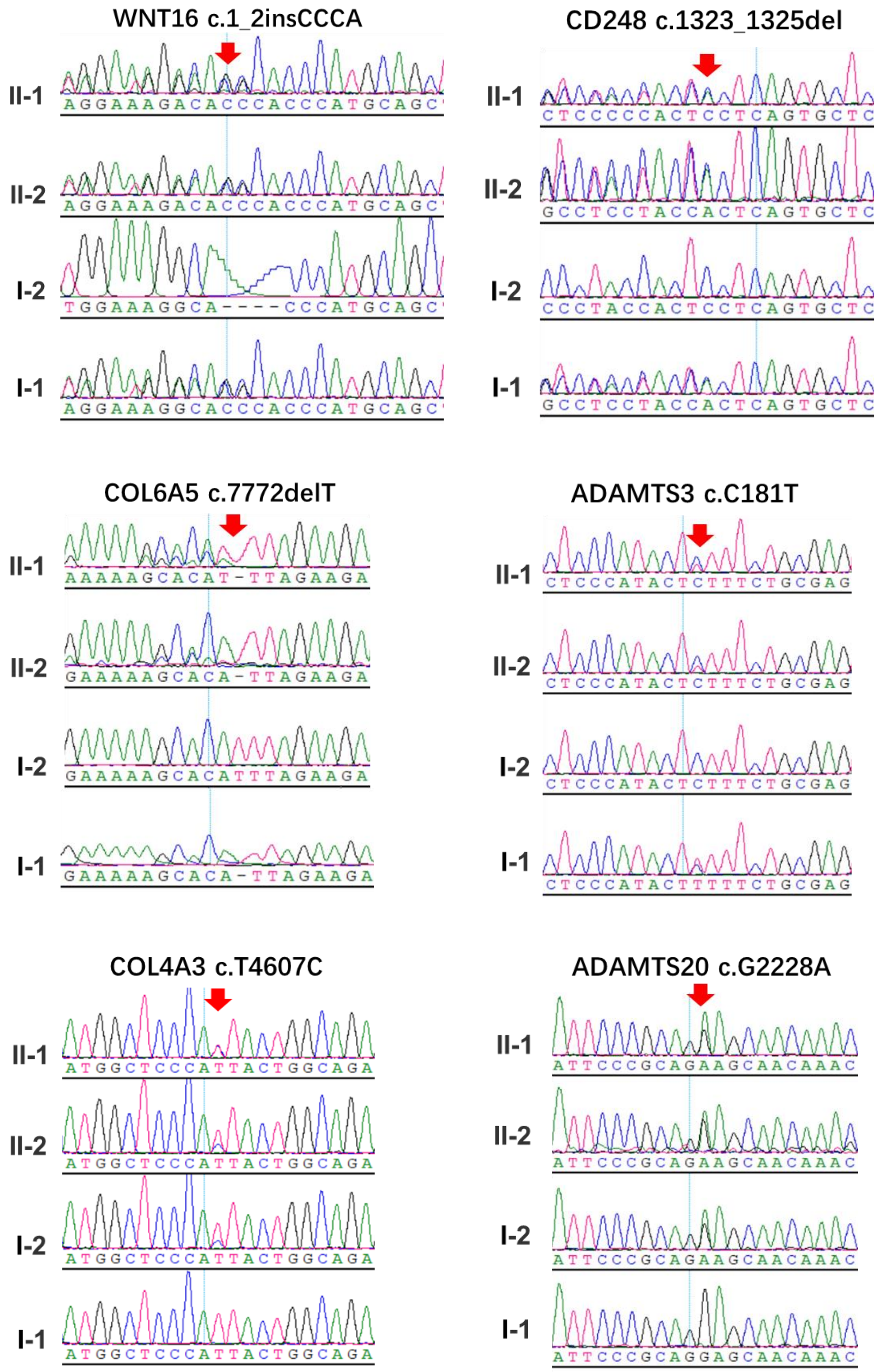

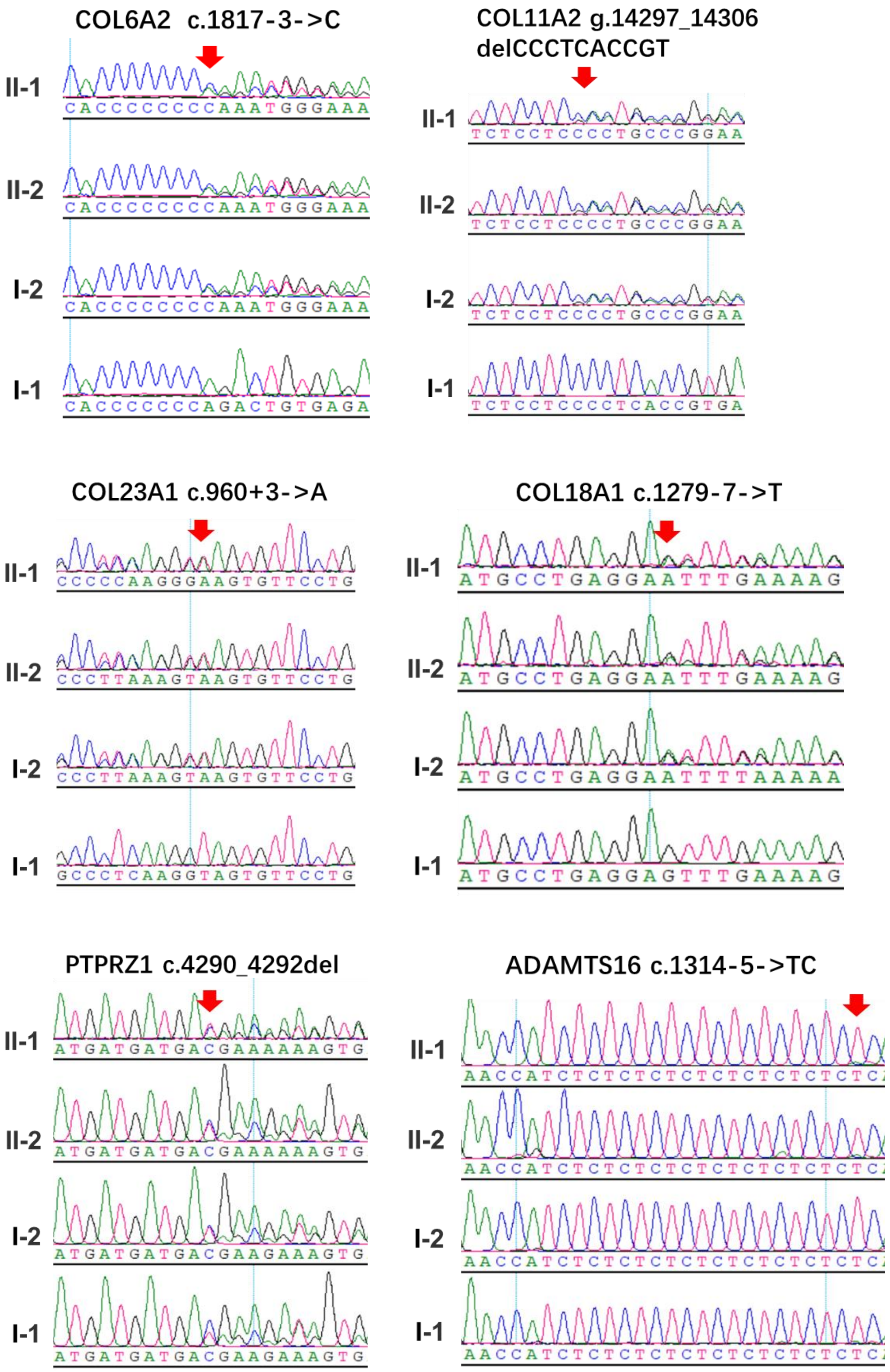
COL6A2 c.1817-3->C
II-1
CACCCCCCCCAAATGGGAAA
II-2
CACCCCCCCCAAATGGGAAA
I-2
CACCCCCCCCAAATGGGAAA
I-1
CACCCCCCCAGACTGTGAGA
COL11A2 g.14297_14306 delCCCTCACCGT
II-1
TCTCCTCCCCTGCCCGGAA
II-2
TCTCCTCCCCTGCCCGGAA
I-2
TCTCCTCCCCTGCCCGGAA
I-1
TCTCCTCCCCTCACCGTGA
COL23A1 c.960+3->A
II-1
CCCCCAAGGGAAGTGTTCCTG
II-2
CCCTTAAAGTAAGTGTTCCTG
I-2
CCCTTAAAGTAAGTGTTCCTG
I-1
GCCCTCAAGGTAGTGTTCCTG
COL18A1 c.1279-7->T
II-1
ATGCCTGAGGAATTTGAAAAG
II-2
ATGCCTGAGGAATTTGAAAAG
I-2
ATGCCTGAGGAATTTTAAAAA
I-1
ATGCCTGAGGAGTTTGAAAAG
PTPRZ1 c.4290_4292del
II-1
ATGATGATGACGAAAAAAGTG
II-2
ATGATGATGACGAAAAAAGTG
I-2
ATGATGATGACGAAGAAAGTG
I-1
ATGATGATGACGAAGAAAGTG
ADAMTS16 c.1314-5->TC
II-1
AACCATCTCTCTCTCTCTCTCTCTCTC
II-2
AACCATCTCTCTCTCTCTCTCTCTCTC
I-2
AACCATCTCTCTCTCTCTCTCTCTCTC
I-1
AACCATCTCTCTCTCTCTCTCTCTCTC

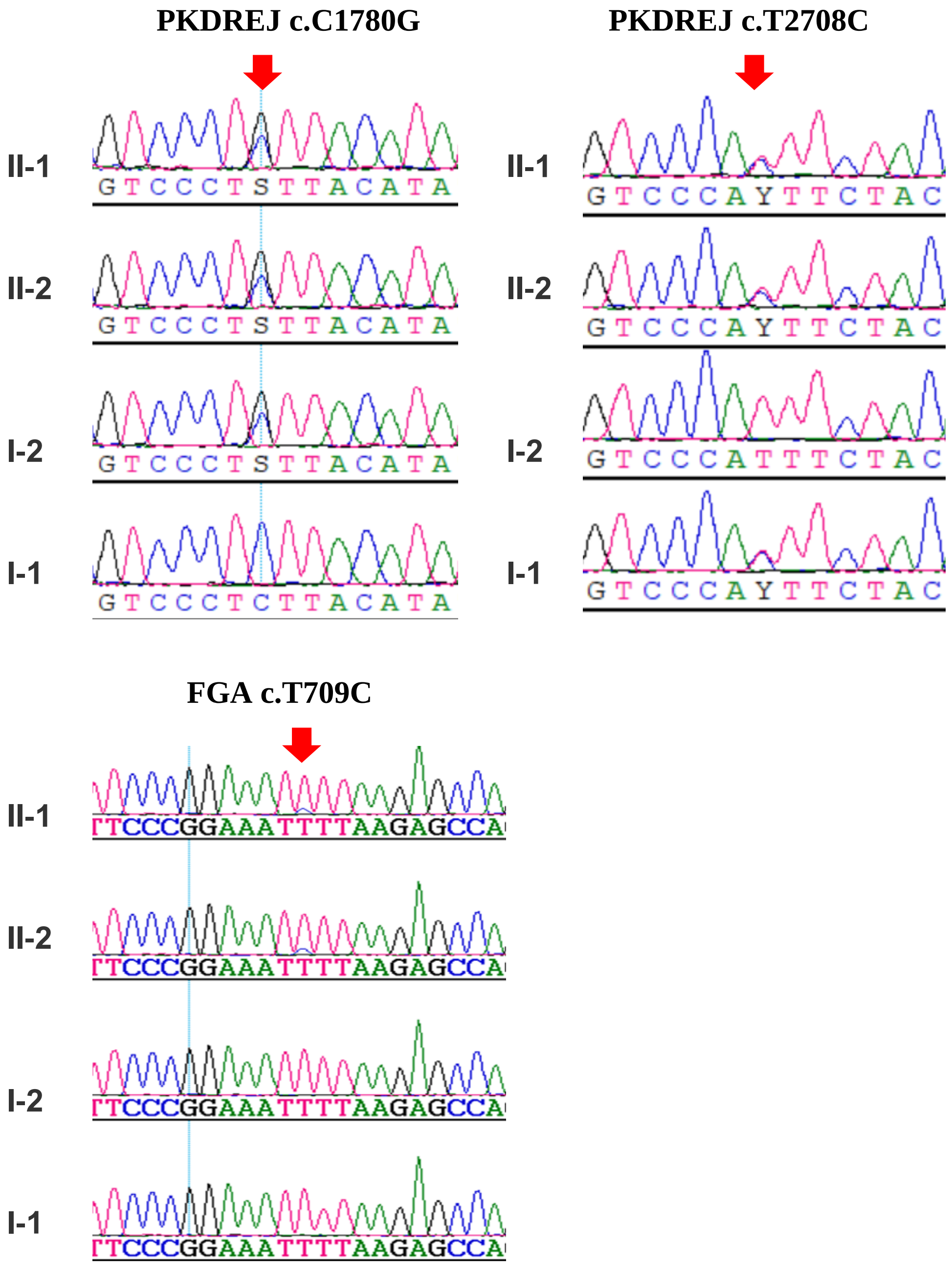

PKDREJ c.C1780G
II-1
GTCCCTSTTACATA
II-2
GTCCCTSTTACATA
I-2
GTCCCTSTTACATA
I-1
GTCCCTCTTACATA
PKDREJ c.T2708C
II-1
GTCCCAYTTCTAC
II-2
GTCCCAYTTCTAC
I-2
GTCCCATTTCTAC
I-1
GTCCCAYTTCTAC
FGA c.T709C
II-1
TTCCCGGAAATTTTAAGAGCCA
II-2
TTCCCGGAAATTTTAAGAGCCA
I-2
TTCCCGGAAATTTTAAGAGCCA
I-1
TTCCCGGAAATTTTAAGAGCCA
