# Supplementary material for: Multi-level consistent changes of the ECM pathway identified in a typical keratoconus twin’s family by multi-omics analysis
Source: Orphanet J Rare Dis. 2020 Aug 31;15:227. doi: 10.1186/s13023-020-01512-7 (PMC7457807; doi:10.1186/s13023-020-01512-7)

**Figure S2.** Protein interaction network between candidate variant genes (inner ring) and differential expression genes (out ring) in shared enrichments.

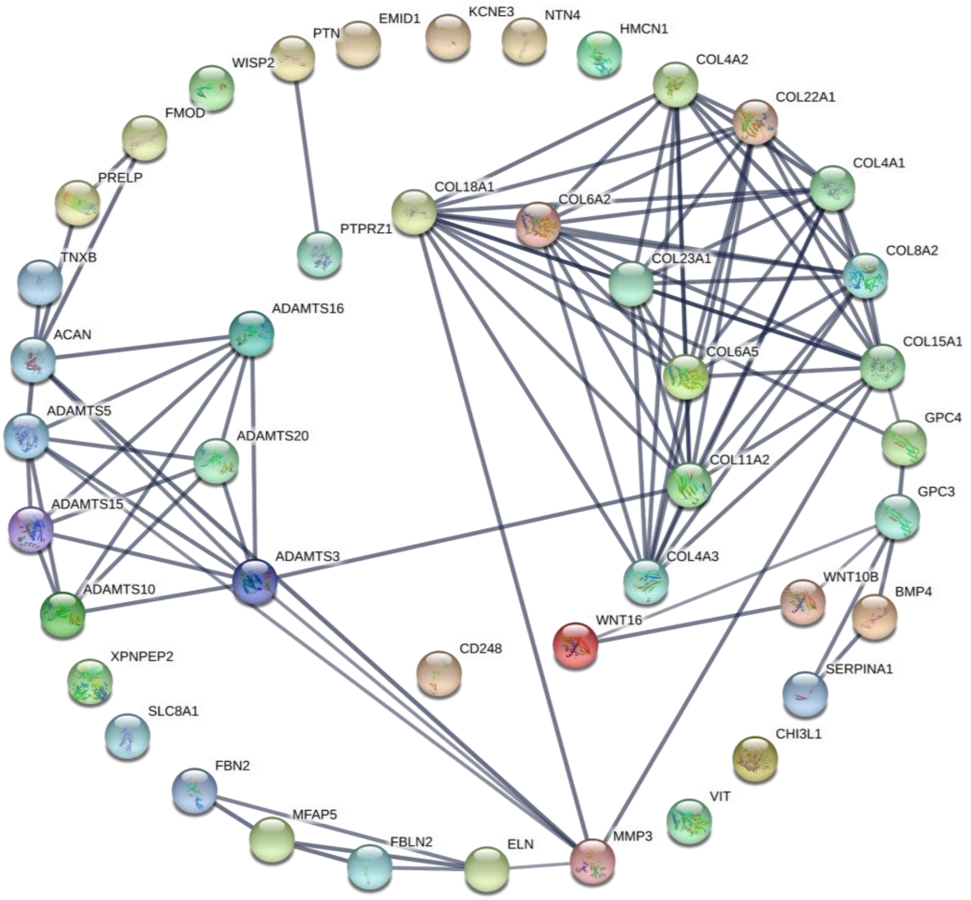

Supplement: Supplementary file 3 — Additional file 3: Figure S2. Protein interaction network between candidate variant genes (inner ring) and differential expression genes (outer ring) in shared enrichments. [file 13023_2020_1512_MOESM3_ESM.pdf]
